# Measurement invariance and cross-linguistic validation of the PSS-4 in university context: multidimensional analysis and associations with psychological and behavioral outcomes

**DOI:** 10.3389/fpsyg.2025.1648070

**Published:** 2025-09-25

**Authors:** Cristina Cañete-Massé, Virginia Krieger, Maribel Peró-Cebollero, Juan Antonio Amador-Campos, Joan Guàrdia-Olmos

**Affiliations:** 1 Departament de Psicologia Social i Psicologia Quantitativa, Secció Psicologia Quantitativa, Facultat de Psicologia, Universitat de Barcelona, Barcelona, Spain; 2 Institut de Neurociencies, Universitat de Barcelona, Barcelona, Spain; 3 Departament de Psicologia Clínica i Psicobiologia, Secció Personalitat, avaluació i tractament psicològic, Facultat de Psicologia, Universitat de Barcelona, Barcelona, Spain; 4 Institut de Sistemes Complexos (UBICS), Universitat de Barcelona, Barcelona, Spain

**Keywords:** university students and staff, psychometric validation PSS-4, Catalan, Spanish, English

## Abstract

**Background:**

Although prior research supports the use of the Spanish version of the PSS-4 using classical psychometric methods, further analysis of its dimensionality, reliability, and response patterns is warranted. Sociodemographic factors such as gender and health behaviors (e.g., sleep, diet, physical activity) may influence perceived stress.

**Objectives:**

This study aimed to: (1) evaluate the reliability and validity of the PSS-4 in Spanish, English, and, for the first time, Catalan; (2) test measurement invariance across language, university groups, and gender; and (3) examine associations between stress and psychological (depression, anxiety, wellbeing) and behavioral outcomes (sleep, internet use, physical activity).

**Methods:**

Participants included 1,810 students and 1,060 university staff, who completed surveys in Spanish, Catalan, or English. Demographic data included gender identity, marital status, education, and lifestyle behaviors. Measures included the PSS-4, WHO-5 Wellbeing Index, GAD Questionnaire, and PHQ. Dimensionality was examined using PCA, followed by invariance testing. The English group comprised a comparatively smaller sample.

**Results:**

The PSS-4 showed a unidimensional structure, high reliability, and strong correlations with psychological outcomes. Measurement invariance was supported at the configural and metric levels but not at the scalar level across languages, university communities, and gender.

**Conclusion:**

The PSS-4 demonstrates validity and reliability for assessing perceived stress in Spanish, English, and Catalan university settings, with this study providing the first validation of the Catalan version and a cross-language invariance test. However, the absence of scalar invariance limits the comparability of stress mean scores across languages.

## Introduction

Stress is an unavoidable experience that is part of the normal pressures of everyday life and it’s a key variable for human health. Research has emphasized the long-term implications of stress on mental health outcomes, psychological wellbeing, and tear on quality of life (Charles et al., 2013; Seo et al., 2018). Lazarus and Folkman (1984) define psychological stress as “a particular relationship between the person and the environment that is appraised by the person as taxing or exceeding his or her resources and endangering his or her wellbeing” (p.19). Thus, although stress is not always a negative experience (Folkman, 2013), it is the subjective perception of the uncontrollability and unpredictability of stressors, along with one’s personality, coping resources, and support factors, that determines whether coping strategies are initiated and whether the stressor is ultimately resolved (Biggs et al., 2017; Phillips, 2020).

Perceived stress in adults may be linked to several life outcomes, including psychological outcomes (i.e., depression and anxiety) (Fassett-Carman et al., 2020; Seo et al., 2018), social adjustment difficulties (Yildirim and Green, 2023) and challenges in maintaining a work-life balance (Xu and Wang, 2023). Researchers have also confirmed that female suffer higher levels of stress response than male, with more prevalent emotional exhaustion (Costa et al., 2021; Graves et al., 2021). Recent research has confirmed links between health-related behaviors like physical activity and healthy diet with perceived stress (Bremner et al., 2020; Wright et al., 2023). Moreover, the hours connected to online social interactions seems also a key variable that may influence stress (Tibbetts et al., 2021). Additionally, psychological stress is becoming an established risk factor for academic, social, and personal adjustment challenges faced by university students (Asif et al., 2020; Harris et al., 2023; Trigueros et al., 2020). Research also suggests that university staff experience high stress levels due to job demands and workloads (Jayman et al., 2022; Mark and Smith, 2012). All in all, it is well-demonstrated that stress is influenced by sociodemographic factors (i.e., gender and occupation), health behaviors and internalizing psychopathology.

Since life outcomes are potentially sensitive to factors such as stress, it is imperative to reliably and validly assess an individual’s perception of their overall stressfulness and their ability to handle it (Lee, 2012; Phillips, 2020). From a psychological assessment perspective, stress responses are commonly measured using specific self-report scales (e.g., Crosswell and Lockwood, 2020; Harris et al., 2023), such as the Perceived Stress Scale, which allows for both accurate measurement and the definition of specific intervention targets (Chan and La Greca, 2020). In this framework, the Perceived Stress Scale (PSS; Cohen et al., 1983), has become one of the most widely used tools to assess the effects of stress in both clinical and non-clinical populations (Harkness and Monroe, 2016; Harris et al., 2023; Taylor, 2015). The PSS measures “the degree to which respondents found their lives unpredictable, uncontrollable, and overloading (Cohen et al., 1983, p. 387) in the past month, on a five-point Likert scale ranging from “never” to “very often.” It is available in three standard versions: the 14-item scale (PSS-14), the 10-item scale (PSS-10), and the short form 4-item scale (PSS-4). The shorter scale includes items 2, 6, 7, and 14 from the original scale and was designed for situations with time constraints on data collection and large samples (Cohen et al., 1983). The PSS-14 and PSS-10 scales have been translated, adapted, and validated in multiple languages, including Spanish (Lee, 2012; Maroufizadeh et al., 2018; Schneider et al., 2020). The PSS-4 scale, on the other hand, has only been adapted into a few languages, for instance, Spanish (Vallejo et al., 2018), French (Lesage et al., 2012) or Swedish (Rozental et al., 2023). Additionally, it’ has been used in different populations, including graduate and postgraduate students, workers or teachers, among others (Lee, 2012; Schmalbach et al., 2025).

An extensive body of research has confirmed the PSS’s good internal consistency and test–retest reliability across a wide range of samples and linguistic backgrounds (Lee, 2012; Soares et al., 2020; Warttig et al., 2013). Studies analyzing the psychometric properties of the PSS show that in general PSS-14 and PSS-10 exhibited adequate to high internal consistency reliability, as measured by Cronbach’s alpha (α > 0.70) and test-retest reliability (Pearson’s, Spearman’s or intraclass correlation coefficient > 0.70) (Lee, 2012; Rozental et al., 2023; Taylor, 2015). The results for the PSS-4 are mixed; some studies report relatively low Cronbach’s alphas (α < 0.70) (Lee, 2012), while others report high Cronbach’s alphas and omega coefficients (α = 0.82 and ω = 0.78) (Mitchell et al., 2008; Ruisoto et al., 2020). Furthermore, studies conducted with Spanish-speaking samples have reported acceptable reliability of the PSS-4 (α = 0.70 and α = 0.73; Sanabria-Mazo et al., 2024; Vallejo et al., 2018).

Regarding the factorial structure, studies indicate that for the PSS-14 and PSS-10, a bifactor structure representing positive and negative stress items is more dominant than a one-factor structure (e.g., Hore-Lacy et al., 2024; Reis et al., 2019). Studies analyzing the factor structure of the PSS-4 have yielded mixed results. While some studies suggest that an unifactorial model is inadequate (Mondo et al., 2021), others support it as a viable alternative with satisfactory statistical properties (Lee, 2012; Lesage et al., 2012; Ruisoto et al., 2020). It is important to highlight that the consistent identification of a one-factor structure in the PSS-4 is likely due to the reduced number of items, which limits the detection of multiple dimensions, and the limited variation in item wording effects (Lee, 2012; Lesage et al., 2012). Furthermore, the translation and back-translation process, as well as the characteristics of the sample, can reinforce a unified perception of stress, which, together with the use of brief administration formats (e.g., online), may support a one-dimensional structure (e.g., Lee, 2012; Lesage et al., 2012; Ruisoto et al., 2020).

Furthermore, regarding measurement invariance (MI), evidence suggests high MI across gender for the PSS-10 (Reis et al., 2019) and configural, metric, and scalar invariance between groups according to sociodemographic variables such as income level and work status for the PSS-4 (Sanabria-Mazo et al., 2024). These results may help to understand the importance of assessing dimensionality of PSS in different groups and languages.

Although previous psychometric studies of the PSS-4 in Spanish using classical psychometric approaches have contributed to its widespread use, its dimensionality requires further evaluation. We need to assess its reliability and identify any potential response patterns that could impact the scores. In this sense, sociodemographic variables as gender along with health-related behaviors like physical activity, hours of sleep, and type of diet, could influence the perceived stress. Moreover, examining the convergent validity with other psychological outcomes could highlight the protective and risk factors of stress, helping to assess it before it appears.

In light of the results of the aforementioned studies, we hypothesized that the PSS-4, administered in three languages and evaluated using the classical psychometric approach, will show reliability and validity. In this sense, Spanish and English versions have already demonstrated validity and reliability (Vallejo et al., 2018; Warttig et al., 2013) but it is the first time to validate the Catalan version of the PSS-4. We also hypothesized that measurement invariance would be met across language, university community, and gender, as these factors may be key to ensuring fair comparisons between groups, enhancing the validity of the results, and improving the precision of psychological assessment. Only one study (Sanabria-Mazo et al., 2024) has studied measurement invariance in a Colombian sample using the PSS-4 with sociodemographic information, and they found measurement invariance for gender and age, but not for income level and working status. It is relevant to study the results with a Spanish and English language, as well as for Catalan. Additionally, we hypothesized that the PSS-4 scale would show validity through its expected associations with both traditional psychological outcomes (i.e., depression, anxiety, and wellbeing) and health-related behaviors (i.e., hours of sleep, time spent online, and physical activity).

## Materials and methods

### Participants

The present study was part of a bigger project which aimed to study the mental health and the emotional wellbeing of the students and workers of the Catalonian universities. The sample was comprized of 2.870 participants of the University of Barcelona., 1.810 university students (mean age = 22.91, SD = 6.6; 74.8% females, 21.7% males, 2.4% non-binary, 0.2% others, and a 1.0% preferred not to answer). A 55.5% answered the survey in Spanish, a 39.8% in Catalan and a 4.6% in English) and 1,060 university staff (mean age = 47.21, SD = 11.38; 64.9% females, 32.8% males, 1.2% non-binary and 1.0% preferred not to answer. A 31.2% answered in Spanish, a 65.1% in Catalan and a 3.7% in English).

The inclusion criteria required participants to be over 18 years of age, affiliated with the University of Barcelona as either students or university staff, to be speakers of one of the three target languages (i.e., Catalan, Spanish, or English), and to have fully completed one or more of the measures included in the online survey.

### Instruments

*Ad hoc* questionnaires were developed to assess age, gender, marital status, university community, education level, physical activity, sleeping, and diet, measured through the following question:

- How many hours of physical activity do you do in a week? 30 min or less/1–2/3–4/5 h or more.
- How many hours do you sleep on average every day? 5 h or less, 6, 7, 8, 9 h or more.
- How many hours do you connect every day to the internet (mobile, PC, tablet, smart watch) for purposes other than working or studying? Between 0 and 1 h, between 1 and 2 h, between 3 and 4 h, between 4 and 5 h, more than 5 h.
- Do you think your diet is healthy? Yes/No.

Perceived Stress Scale (PSS-4; Cohen et al., 1983): short version of the PSS-10, with 4-item self-report questionnaire to assess stress. Each item is rated on a five-point Likert scale (0 = never, 1 = Almost never, 2 = Sometimes, 3 = Fairly often, 4 = Very often). The score ranges from 0 to 16. The PSS-4 has no clinical cut-off scores, so individual scores are compared with normative values (averaged stress scores ≥ 6), with higher scores reflecting higher levels of perceived stress (Warttig et al., 2013). In this study, the Cronbach’s alpha of this questionnaire was 0.802 indicating good reliability.

Before data collection, two clinical psychologist investigators with training and experience in psychological assessment, as well as in the translation, adaptation, and validation of questionnaires translated, the original English version of the PSS-4 and the four equivalent items of the Spanish version into Catalan. A trilingual (English, Catalan and Spanish) expert panel reviewed the Catalan translation of the PSS-4 and provided detailed and comprehensive feedback. The committee was composed of two psychologists with experience in mental health and public health researchers. An investigator of the team compiled and synthesized the feedback provided by the committee members and drafted the final Catalan version of the PSS-4. All discrepancies were discussed by the panel and resolved until they reached a formal consensus. Subsequently, a pilot test of the first Catalan version of the PSS-4 was distributed to a selected sample of students and university staff, who were invited to provide comments and suggestions regarding the clarity and comprehensibility of the items. The three versions (Catalan, English, and Spanish) used in this study are presented in Supplementary Table 1.

Five Wellbeing Index (WHO-5; Bech, 2004). The WHO-5 is a five-item self-report questionnaire to assess subjective psychological wellbeing (Topp et al., 2015). Each item is rated on a five-point Likert scale (0 = at no time, 1 = Some of the time, 2 = less than half of the time, 3 = more than half of the time, 4 = Most of the time, 5 = All of the time). The total score ranges from 0 to 25, and a score below 13 may suggest poor wellbeing. In this study, Cronbach’s alpha of this questionnaire was 0.883 indicating good reliability.

Patient Health Questionnaire (PHQ-9, Diez-Quevedo et al., 2001; Spitzer et al., 1999): The PHQ-9 includes 9 items designed to screen for depression symptoms based in DSM-IV criteria for depressive disorders. Each item is rated on a four-point Likert scale (0 = none, 1 = less than half the time, 2 = more than half the time, and 3 = almost every day) with total scores ranging from 0 to 27. Higher scores indicate more depressive symptoms. In this study, the Cronbach’s alpha of this questionnaire was 0.881 indicating good reliability.

Generalized Anxiety Disorder Questionnaire (GAD-7; Martínez-Vázquez et al., 2022; Spitzer et al., 2006): The GAD-7 is a seven-item self-report scale designed to assess anxiety symptoms. Each item is rated on a four-point Likert scale (0 = not at all, 1 = several days, 2 = more than half the days, 3 = nearly every day), with total scores ranging from 0 to 21. Higher scores indicate more anxiety symptoms. In this study, the GAD-7 demonstrates excellent internal reliability, with a Cronbach’s alpha of 0.915.

### Procedure

The present project was approved by the ethics committee of the Catalonian universities. All the students and university staff received a link to the online survey sent by the vice-rectorate of the University. The whole assessment was performed online using Qualtrics and the data recollection was performed from 27 March 2023 until 28 April 2023. The informed consent was obtained by all the participants in the same survey. The participants could answer the survey in Catalan, Spanish, or English. Participation was voluntary and participants did not receive financial compensation for their participation. Questionnaires that contained incomplete responses to key items, or that were duplicated, or that exhibited straight-line answering patterns were excluded. Specifically, participants with missing data on all four PSS items were not considered in the analysis for the current study. In total, 461 questionnaires from students and 281 from university staff were removed due to missing responses on the PSS-4. No imputation was performed for missing data in this instrument. We did not assess baseline differences between included and excluded participants, as those who were excluded due to incomplete PSS-4 responses had not provided sufficient data on the variables of interest t, because most of them only entered to the questionnaire and did not answer any question. As such, potential selection bias could not be formally evaluated. As such, potential selection bias could not be formally evaluated. This study was carried out in accordance with the Helsinki Declaration on Ethical Principles for Medical Research Involving Human Subjects, approved by the Ethics Committees of all the Catalan universities.

### Data analysis

Firstly, the construct validity of the PSS-4 was tested using Principal Component Analysis (PCA). Given the extremely small number of items (only four), PCA is more appropriate than Exploratory or Confirmatory Factor Analysis (EFA/CFA), which require a larger number of variables to produce stable and meaningful factor structures (Fabrigar et al., 1999; MacCallum et al., 1999). PCA serves as a descriptive data reduction technique that can offer preliminary insights into dimensionality without imposing strong model assumptions (Costello and Osborne, 2005). EFA and CFA involve latent variable modeling and error estimation, which are not feasible or theoretically justified with such a limited number of observed variables (MacCallum et al., 1999). Therefore, PCA is a pragmatic choice for assessing unidimensionality in short scales consisting of very few items (Fabrigar et al., 1999). Moreover, as the structure has already been assessed, we want to confirm the unidimensional structure. In addition to the estimation of the PCA, the factor loadings and their 95% confidence intervals (CI) have been also calculated using a the percentile bootstrap with 1,000 samples. The reliability was tested using Cronbach’s alpha and McDonald’s ω. The principal aim of this analysis was to demonstrate the unidimensional structure as it has been demonstrated in other studies: Spanish (Vallejo et al., 2018) and English (Warttig et al., 2013). In this case, it is the first time that a validation of the PSS-4 is presented in Catalan language, so it is important to assess the construct validity and reliability as key psychometric analyses. The one-dimensionality of the scale and the reliability had to be demonstrated for each language, therefore, the analyses were performed separately for each language: for the Spanish validation, a total sample of *n* = 1,336 was used. For Catalan version, a total of *n* = 1,411 was used, and finally, for English version, a total of *n* = 123 was used.

Once the one-dimensionality of the scale was demonstrated, measurement invariance was assessed. In this sense, only one very recent Colombian validation (Sanabria-Mazo et al., 2024) has tested measurement invariance regarding sociodemographic characteristics. It is crucial to study measurement invariance in a Spanish population to guarantee fair comparisons between groups and to increase the results validity, as well as to increase the precision of the psychological evaluation. This analysis was performed for languages (Spanish, *n* = 1,336; Catalan, *n* = 1,411; English, *n* = 123), for community (1,810 university students and 1,060 university staff members) and for gender (1,353 females and 541 men).

As the configural and the metrical invariance was confirmed, the next step was to demonstrate the relationships between PSS-4 and other psychological outcomes and health-related behaviors. The relationships between the PSS-4 and anxiety and depression have already been demonstrated in the Spanish version (Vallejo et al., 2018). In this regard, the aim of this study was to explore the relationships of stress with both anxiety and depression, as well as with psychological wellbeing and health-related behaviors (type of diet, number of hours of sleep, number of hours of physical activity, number of hours connected to the internet outside of working/studying hours). This analysis was performed for the whole sample, as it has been mentioned above, once the configural and the metrical invariance is accomplished, the relationship between variables can be compared. In this case, correlations were performed for the quantitative variables, whereas ANOVAS with Tuckey *post hoc* comparisons were performed for the multiple answer questions.

All the analysis were performed with R studio, using lavaan package (Rosseel, 2012) and psych (Revelle, 2025) for the bootstrap estimation in the CI.

## Results

### Structural validity and reliability

For the Spanish version (*n* = 1,336), PCA was carried out to determine the factor structure of PSS-4. Both the Kaiser-Meyer-Olkin test (0.787) and Barlett’s test of sphericity (χ^2^ = 1807.218; *df* = 6; *p* < 0.001) indicate that a reduction in dimensionality is appropriate. On the other hand, the scree plot indicates that a single component with an eigenvalue of 2.55 is a good solution and therefore explains 63.9% of the total variability of the scale.

For the Catalan version (*n* = 1,411), PCA was carried out to determine the factorial structure of PSS-4. Both the Kaiser-Meyer-Olkin test (0.784) and Barlett’s test of sphericity (χ^2^ = 2123.910; *df* = 6; *p* < 0.001) indicate that a reduction in dimensionality is appropriate. On the other hand, the scree plot indicates that a single component with an eigenvalue of 2.61 is a good solution and therefore explains 65.4% of the total variability of the scale.

For the English version (*n* = 123), PCA was carried out to determine the factor structure of PSS-4. Both the Kaiser-Meyer-Olkin test (0.693) and Barlett’s test of sphericity (χ^2^ = 110.49; *df* = 6; *p* < 0.001) indicate that a reduction in dimensionality is appropriate. On the other hand, the scree plot indicates that a single component with an eigenvalue of 2.20 is a good solution and therefore explains 55.5% of the total variability of the scale. Table 1 presents the factor loadings and associated standard errors for each item across languages.

**TABLE 1:** Factor loadings and their confidence interval for each item for each language.

Languages	Catalan	English	Spanish
Item 1 FL (95% CI)	0.823 (0.800–0.844)	0.730 (0.547–0.831)	0.823 (0.803–0.841)
Item 2 FL (95% CI)	0.760 (0.727–0.789)	0.655 (0.438–0.790)	0.753 (0.718–0.786)
Item 3 FL (95% CI)	0.803 (0.782–0.822)	0.757 (0.655–0.830)	0.802 (0.778–0.823)
Item 4 FL (95% CI)	0.846 (0.829–0.861)	0.817 (0.749–0.873)	0.817 (0.796–0.836)

FL, factor loading; 95% CI, percentile bootstrap 95% confidence interval in 1,000 samples.

Table 2 presents the reliability measures for the three languages. As it can be seen, the three languages present good internal consistency following Prinsen et al. (2018).

**TABLE 2:** Measures of reliability for each language.

Language	McDonald’s ω (95% CI)	Cronbach’s α (95% CI)
Spanish version	0.814 (0.794–0.832)	0.810 (0.793–0.825)
Catalan version	0.829 (0.811–0.845)	0.821 (0.806–0.835)
English version	0.735 (0.628–0.813)	0.724 (0.637–0.794)

CI, confidence interval.

### Measurement invariance

Table 3 summarizes the results of the measurement invariance for language. As it can be seen, the metric model is accomplished (no significant differences with the configural model). This means that the items can be grouped in the same factorial structure and that the factorial loadings are equal across languages, allowing for comparisons between variables. However, there are significant differences between the metric and the scalar model. This means that the intercepts are not equal and therefore, mean comparisons between groups are not strictly comparable.

**TABLE 3:** Measurement invariance for language.

Model	χ^2^ (df)	CFI	RMSEA	SRMR	ΔX^2^	*P* (ΔX^2^)
Configural	50.48 (6)	0.966	0.087	0.026	–	–
Metric	56.84 (12)	0.966	0.06	0.030	6.36	0.3839
Scalar	102.63 (18)	0.936	0.07	0.034	45.78	< 0.001

χ^2^, chi squared; CFI, comparative fit index; RMSEA, root mean square error of approximation; SRMR, Standardized Root Mean Square Residual.

Table 4 summarizes the results of the measurement invariance for university communities. As it can be seen, the metric model is accomplished (no significant differences with the configural model). However, there are significant differences between the metric and the scalar model. Similarly, as in language, the intercepts are not equal and therefore, mean comparisons between groups are not strictly comparable.

**TABLE 4:** Measurement invariance for university communities.

Model	χ^2^ (df)	CFI	RMSEA	SRMR	ΔX^2^	*P* (ΔX^2^)
Configural	50.085 (4)	0.966	0.09	0.032	–	–
Metric	55.704 (7)	0.960	0.07	0.033	5.6192	0.131
Scalar	77.618 (10)	0.944	0.069	0.037	21.914	< 0.001

χ^2^, chi squared; CFI, comparative fit index; RMSEA, root mean square error of approximation; SRMR, Standardized Root Mean Square Residual.

Table 5 summarizes the results of the measurement invariance for gender. As it can be seen, the metric model is accomplished (no significant differences with the configural model). However, there are significant differences between the metric and the scalar model. Analogous to the case of language and university communities, the intercepts are not invariant, implying that mean-level comparisons between groups should be interpreted with caution.

**TABLE 5:** Measurement invariance for gender.

Model	χ^2^ (df)	CFI	RMSEA	SRMR	ΔX^2^	*P* (ΔX^2^)
Configural	52.803 (4)	0.963	0.093	0.027	–	–
Metric	53.12 (7)	0.965	0.068	0.027	0.317	0.956
Scalar	69.74 (10)	0.955	0.065	0.031	16.62	< 0.001

χ^2^, chi squared; CFI, comparative fit index; RMSEA, root mean square error of approximation; SRMR, Standardized Root Mean Square Residual.

### Relationships between PSS-4 and other psychological outcomes and health-related behaviors

This analysis was performed for the whole sample, as noted earlier, once the configural and the metrical invariance is accomplished, the relationship between variables can be compared. It is important to highlight that the sample size differs across questionnaires, as some participants did not complete them in full. Table 6 shows the correlations between PSS-4 and other measures. There were high and significant negative correlations, with a high effect size, between scores of PSS-4 and WHO-5 highlighting an indirect relationship between stress and wellbeing. There were high and significant correlations, with a high effect size, between scores of PSS-4 and scores of PHQ-9 and GAD-7 indicating a direct relationship between stress, anxiety and depression.

**TABLE 6:** Correlations of Perceived Stress Scale (PSS-4) with Five Wellbeing Index (WHO-5), Patient Health Questionnaire (PHQ-9) and Generalized Anxiety Disorder Questionnaire (GAD-7).

	WHO-5	PHQ-9	GAD-7
PSS-4	*r* = −0.673	*r* = 0.707	*r* = 0.649
	*n* = 2,841	*n* = 2,769	*n* = 2,739
	*r*^2^ = 0.453	*r*^2^ = 0.500	*r*^2^ = 0.421
	*p* = < 0.001	*p* = < 0.001	*p* = < 0.001

r, pearson correlation; *n*, sample size; *r*^2^, effect size; p, *p*-value.

Table 7 shows the descriptive statistics regarding stress linked to hours doing physical activity, hours sleeping and hours connecting. Regarding physical activity, significant differences were found in students (F = 27.5; *df* = 3,2866; *p* = < 0.001; ω*^2^* = 0.027), with little effect size. *Post hoc* analyses revealed significant differences between doing 30 min or less of physical activity and the rest of hours (*p_*scheff*é_* < 0.001). Regarding hours sleeping, significant differences were found between workers (F = 35.5; *df* = 4,2865; *p* = < 0.001; ω*^2^* = 0.046), with a little-mean effect size. Post-hoc analyses revealed significant differences between students sleeping 5 h or less and 6 h (*p_*scheff*é_* < 0.001), 7 h (*p_*scheff*é_* < 0.001), and 8 h (*p_*scheff*é_* < 0.001). There were also significant differences between students sleeping 6 and 7 h (*p_*scheff*é_* < 0.001) and 8 h (*p_*scheff*é_* < 0.001). Regarding hours connected to the internet, significant differences were found (F = 26.3; *df* = 5,2864; *p* = < 0.001; ω*^2^* = 0.042), with little-mean effect size. *Post hoc* analyses revealed significant differences between stay connected 0–1 and 1–2 h (*p_*scheff*é_* = 0.015); 2–3 h (*p_*scheff*é_* < 0.001), 3–4 h (*p_*scheff*é_* < 0.001), 4–5 h (*p_*scheff*é_* < 0.001) and more than 5 h (*p_*scheff*é_* < 0.001); between stay connected 1–2 h and the rest of categories (*p_*scheff*é_* < 0.001) except 2–3 h; between stay connected 2, 3, and 4–5 h (*p_*scheff*é_* = 0.009) and more than 5 h (*p_*scheff*é_* = 0.003). Finally, regarding the type of diet, significant differences were found in students between those who thought they had a healthy diet and those who not (*t* = 11.90, *df* = 2,868; *p* < 0.001; *Cohen’s d* = 0.523; ${\bar{x}}_{h\,e\,a\,l\,t\,h\,y}$ = 7.12 (*n* = 2,190, SD = 3.19); ${\bar{x}}_{n\,o\,t\,h\,e\,a\,l\,t\,h\,y}$ = 8.79 (*n* = 680, SD = 3.17), indicating a medium effect size.

**TABLE 7:** Descriptives of hours doing physical activity and hours sleeping linked to stress among students and university staff.

Hours doing physical activity	*n*	Mean PSS-4 (SD)
30 min or less	767	8.39 (3.31)
1–2 h	897	7.36 (3.14)
3–4 h	783	7.16 (3.23)
5 h or more	423	6.91 (3.23)
**Hours sleeping**	***n***	**Mean PSS-4 (*SD*)**
5 h or less	292	9.37 (3.32)
6 h	837	7.74 (3.14)
7 h	1163	7.08 (3.22)
8 h	516	6.99 (3.13)
9 h or more	62	8.24 (3.28)
**Hours connected to the internet**	***n***	**Mean PSS-4 (SD)**
Between 0 and 1 h	292	6.14 (3.21)
Between 1 and 2 h	711	6.97 (3.32)
Between 2 and 3 h	626	7.43 (3.13)
Between 3 and 4 h	413	8.04 (3.27)
Between 4 and 5 h	347	8.27 (3.13)
More than 5 h	481	8.26 (3.06)

## Discussion

Mental health prevention is crucial in current times, therefore early detection of stress with valid and reliable measures could help to promote positive wellbeing and resilience and consequently, improve the quality of life among adults. In particular, early detection of stress can serve as a protective factor in preventing the onset of stress-related psychopathologies, such as anxiety disorders and depression (Weger and Sandi, 2018). Therefore, regarding the first hypothesis, which proposed that the PSS-4, administered in Spanish, English, and Catalan to a sample of students and university staff, would demonstrate good psychometric properties, the results supported this assumption. Specifically, the PSS-4 demonstrated a clear unidimensional structure across all three language versions, with higher variance explained by one factor than in other studies in Spanish (Vallejo et al., 2018). It should be noted that the one-factor structure observed across the three versions of the PSS-4 may be partly related to the small number of items. When a scale includes fewer items with good internal coherence, it makes it easier for a single dimension to account for a high proportion of the variance. Likewise, both the translation and back-translation process and the use of brief administration formats (i.e., online) may contribute to the emergence of a one-factor structure (e.g., Lee, 2012; Sanabria-Mazo et al., 2024). Additionally, to our knowledge, this is the first time a PCA has been performed on the PSS-4 in Catalan. Regarding reliability, the three version of PSS-4 showed high reliability measured with Cronbach’s α and McDonald’s ω, having similar results as the English version (Warttig et al., 2013). In the Spanish version used in our study, the PSS-4 demonstrated higher reliability compared to the findings reported by Vallejo et al. (2018), Sanabria-Mazo et al. (2024), the latter conducted on a sample of university students.

Concerning the second hypothesis, which proposed that measurement invariance would be established across languages, university community, and gender, the findings offer only partial support. Indeed, as mentioned, the configural and the metrical invariance was met, whereas the scalar invariance was not accomplished for the three variables.

The absence of scalar invariance indicates that mean comparisons of stress scores across language, university communities, and gender should be approached with caution, as the equality of intercepts is a prerequisite for valid group comparisons (Vandenberg and Lance, 2000; Sass, 2011). In this sense, the results support that the items can be grouped in the same factorial structure (configural invariance), and that the factor loadings are equivalent across groups (metrical invariance). These levels of invariance support the analysis of relationships between variables. However, the lack of scalar invariance, which reflects unequal intercepts, limits the interpretability of mean-level comparisons. In the case of language, several factors may underlie this non-invariance, despite it is the first time to our knowledge that has been assessed. For instance, linguistic differences may alter how items are interpreted, since translations often fail to capture cultural nuances in the expression of stress (Cruz-Gonzalez et al., 2021). In the case of university communities, another time it is the first time to be assessed. This finding is particularly relevant given that both students and staff within university settings are consistently reported to experience significant levels of stress (La Fauci et al., 2023; Li and Kou, 2018). The lack of scalar invariance may reflect differences in how stress is experienced or reported across these groups. Students are exposed to numerous stressors, including frequent evaluations, high academic workload, and financial difficulties (Asif et al., 2020; Trigueros et al., 2020). University staff, meanwhile, face high demands and pressure to demonstrate competence, as well as challenges related to job satisfaction (Agyapong et al., 2022; Mark and Smith, 2012). Therefore, this may also reflect differing norms regarding what is perceived as stressful, influencing the relationship between items and the latent construct (Byrne and van de Vijver, 2010). Finally, regarding gender, to date, only one study has evaluated the PSS-4 with respect to measurement invariance and gender in a Colombian sample (Sanabria-Mazo et al., 2024), showing inconsistent results with ours, as they found the PSS-4 to be invariant across gender. However, significant differences in stress by gender have been already demonstrated in other studies (Costa et al., 2021; Graves et al., 2021) which could justify this invariance.

The fact that scalar invariance is not supported, while metric and configural invariance are, has some practical and clinical implications that worth mentioning when using the PSS-4 for assessment. In reality, these findings align with prior research showing that psychological measures often fail to achieve full scalar invariance across diverse populations, underscoring the need for cautious interpretation and reliance on invariant items for cross-group comparisons. In practice, the lack of scalar invariance in the PSS-4 across language, university communities, and gender implies that mean comparisons between groups cannot be interpreted unambiguously, as differences may reflect measurement bias rather than true disparities in perceived stress. This limits the validity of using raw total scores to compare groups directly. A pragmatic approach is to explore partial invariance models, where equality constraints are retained for invariant items and relaxed for those showing non-invariance (Putnick and Bornstein, 2016). Such models allow for more accurate latent mean comparisons while retaining the usefulness of the instrument. Alternatively, researchers may focus on relations between stress and external variables, since metric invariance is generally sufficient for comparing structural associations across groups. These strategies enable continued use of the PSS-4 while acknowledging its measurement limitations in cross-group contexts, in other words, clinically, it undermines the validity of cross-group assessments and may lead to unfair or inaccurate conclusions.

Finally, the results related to the third hypothesis, which proposed that the PSS-4 scale would be associated with both traditional psychological outcomes and health-related behaviors, provided only partial support. Precisely, despite the low effect size the PSS-4 showed a strong association with measures of anxiety and depression, consistent with previous findings (Vallejo et al., 2018), indicating a linear relationship with both constructs. Regarding wellbeing, to our knowledge, this study is the first to measure the relationship between this construct and the PSS-4. The findings reveal a strong, indirect relationship between the two measures, with higher stress levels indicating lower personal wellbeing. Thus, the significant and strong correlations observed between PSS-4 scores and measures of anxiety, depression, and well-being are consistent with findings reported in the established literature (Lee, 2012). These results suggest that the PSS-4 effectively captures the core dimensions of perceived stress in relation to these key psychological constructs.

Stress demonstrated to have also relationships with other relevant health-related behaviors. Concerning hours of physical activity, hours of sleep, hours spent connected to the internet, and type of diet variables, statistically significant differences were observed also with very few effect size, in line with other studies (Bremner et al., 2020; Tibbetts et al., 2021; Wright et al., 2023). However, these differences are considered negligible, as indicated by the effect size measures in the four cases and as can be further supported by Table 7, which shows that means are nearly identical across groups.

In summary, we found higher levels of stress to be associated with more mental health problems as well as poorer personal wellbeing.

The results of this study need to be interpreted in light of a number of limitations. First, a key limitation is the use of a cross-sectional design, that makes difficult make a causal inference for the direction of the associations (Wang and Cheng, 2020). Further studies should be conducted on university population using longitudinal approaches to analyze the predictability of relationships and the possible causality between subdimensions. In this regard, Cook et al. (2024) reported adequate model fit supporting a unidimensional structure of the PSS-4 across three timepoints, spaced 6 months apart, over a 1 year period in a sample of 361 mental health counselors.

Additionally, it should be noted that behavioral variables (e.g., sleep, physical activity) may both influence and be influenced by perceived and reported stress. Future research should consider incorporating standardized and validated behavioral instruments to strengthen the findings related to behavioral variables such as sleep, physical activity and sedentary time (i.e., screen activities). Second, given that the pilot testing phase to provide initial item validation was carried out via convenience groups rather than randomized groups, caution is warranted regarding the generalizability of the results (Kalkbrenner, 2021). Third, the relatively small validation sample size of the English group (*n* = 123) compared to the Spanish (*n* = 1,336) and Catalan (*n* = 1,411) groups makes it challenging to accurately understand the nature of the differences between them. The difference in the sample size may reduce the stability of factor loadings and the power of invariance tests in the smaller samples. Nonetheless, the factorial structure was consistent across languages, and the main loadings proved robust. These findings suggest that the observed results are not solely an artifact of sample size, although future studies with larger and more balanced groups would be valuable to confirm them.

Finally, it must be borne in mind that, although the Catalan version of the PSS-4 has shown good psychometric properties, it may contain cultural and linguistic nuances that influence item interpretation. Despite careful efforts to ensure semantic equivalence through translation and back-translation procedures, subtle differences in meaning and cultural context may affect participants’ understanding and responses. Future research is encouraged to further validate the Catalan version using culturally sensitive research methods.

Even so, these limitations, to our knowledge, this is the first study to analyze the psychometric properties of the PSS-4 using a mixed analysis strategy that combines classical psychometric methods with recent theoretical approaches in psychology. Indeed, on the one hand, the classical psychometric approach allowed for accurate structural validity and reliability delimitation of the PSS-4 for university population. Lastly, it is worth highlighting the development and analysis of the Catalan version of PSS-4, which, as far as we know, is being done for the first time.

The findings of this study align with previous research demonstrating the adequate psychometric properties of the PSS-4 in a Spanish-speaking sample (Vallejo et al., 2018). These results are consistent with other recent studies indicating that the PSS-4 is highly reliable and valid for detecting perceived stress levels in adults in a very quick and efficient manner (e.g., Schmalbach et al., 2025).

In summary, this study provides robust evidence supporting the psychometric properties of the PSS-4 in Spanish, English, and Catalan within a university population. The scale demonstrated a consistent unidimensional structure and high reliability. Although configural and metric invariance were established, the absence of scalar invariance suggests meaningful differences in how stress is perceived and reported across groups. From a clinical perspective, these findings underscore the utility of the PSS-4 as a rapid, accessible, and effective screening measure for stress assessment. Its ease of administration makes it particularly suitable for integration into the assessment, prevention, and intervention protocols of university counseling services. Furthermore, the findings emphasize the importance of culturally sensitive assessment approaches in promoting mental health and psychological wellbeing among university population. From a research perspective, the study highlights the need for longitudinal designs, standardized behavioral measures, and further validation of the Catalan version to enhance generalizability and deepen understanding of stress dynamics in diverse academic settings.

## Data Availability

The raw data supporting the conclusions of this article will be made available by the authors, without undue reservation.
