# Correction: Measurement invariance and cross-linguistic validation of the PSS-4 in university context: multidimensional analysis and associations with psychological and behavioral outcomes

**DOI:** 10.3389/fpsyg.2025.1767664

**Published:** 2026-01-12

**Authors:** Cristina Cañete-Massé, Virginia Krieger, Maribel Peró-Cebollero, Juan Antonio Amador-Campos, Joan Guàrdia-Olmos

**Affiliations:** 1 Departament de Psicologia Social i Psicologia Quantitativa, Secció Psicologia Quantitativa, Facultat de Psicologia, Universitat de Barcelona, Barcelona, Spain; 2 Institut de Neurociencies, Universitat de Barcelona, Barcelona, Spain; 3 Departament de Psicologia Clínica i Psicobiologia, Secció Personalitat, avaluació i tractament psicològic, Facultat de Psicologia, Universitat de Barcelona, Barcelona, Spain; 4 Institut de Sistemes Complexos (UBICS), Universitat de Barcelona, Barcelona, Spain

**Keywords:** university students and staff, psychometric validation PSS-4, Catalan, Spanish, English

In the published article, author [Cristina Cañete-Massé] was erroneously spelled as [Cristina Cañete Massé].

The author contributions statement was erroneously given as [CM: Conceptualization, Data curation, Formal analysis, Methodology, Writing – original draft, Writing – review & editing]. The correct author contributions statement is [CC-M: Conceptualization, Data curation, Formal analysis, Methodology, Writing – original draft, Writing – review & editing.].

The original version of this article has been updated.

