# Supplementary material for: Measurement invariance and cross-linguistic validation of the PSS-4 in university context: multidimensional analysis and associations with psychological and behavioral outcomes
Source: Front Psychol. 2025 Sep 25;16:1648070. doi: 10.3389/fpsyg.2025.1648070 (PMC12507836; doi:10.3389/fpsyg.2025.1648070)
Supplement: Supplementary file 1 [file Table_1.docx]

**Supplementary material**

**Supplementary table 1S. Items used for the three versions of the PSS-4 scale**

| Language, prompt and answers | Item 1 | Item 2 | Item 3 | Item 4 |
| --- | --- | --- | --- | --- |
| Catalan: En l'últim mes, amb quina freqüència...  Mai/Gairabé mai/ De tant en tant/ Sovint/ Molt sovint | T'has sentit incapaç de controlar les coses importants de la teva vida | Has estat segur/a sobre la teva capacitat per gestionar els problemes personals | Has sentit que les coses et van bé | Has sentit que les dificultats s'acumulen tant que no les pots superar |
| English: In the last month, how often have you...  Never/ Almost never/ Sometimes/ Farly often/ Very often | Felt that you were unable to control the important things in your life? | Felt confident your ability to handle your personal problems? | Felt that things were going your way? | Felt difficulties were piling up so high that you could not overcome them? |
| Spanish: En el último mes, con qué frecuencia...  Nunca/ Casi nunca/ De vez en cuando/ A menudo/ Muy a menudo | Te has sentido incapaz de controlar las cosas importantes en tu vida | Has estado seguro/a sobre tu capacidad para manejar tus problemas personales | Has sentido que las cosas te van bien | Has sentido que las dificultades se acumulan tanto que no puedes superarlas |
